# Emergence of Mobilized Colistin Resistance Gene *mcr-8.2* in Multidrug-Resistant Enterobacter cloacae Isolated from a Patient in China

**DOI:** 10.1128/spectrum.01217-22

**Published:** 2022-06-21

**Authors:** Siheng Wang, Xiaoyang Ju, Ning Dong, Ruichao Li, Yan Li, Rong Zhang, Yonglu Huang, Hongwei Zhou

**Affiliations:** a Department of Clinical Laboratory, The Second Affiliated Hospital Zhejiang University School of Medicine, Hangzhou, China; b Department of Medical Microbiology, School of Biology and Basic Medical Science, Medical College of Soochow University, Suzhou, China; c Jiangsu Co-innovation Center for Prevention and Control of Important Animal Infectious Diseases and Zoonoses, College of Veterinary Medicine, Yangzhou University, Yangzhou, People’s Republic of China; Instituto de Higiene

**Keywords:** *mcr-8.2*, *Enterobacter cloacae*, colistin, plasmids, multidrug-resistant

## LETTER

Colistin is considered one of the last-resort antibiotics for the treatment of multidrug-resistant (MDR) pathogens infections (1). Liu et al. (2) reported the emergence of plasmid-mediated colistin-resistance gene *mcr-1* in 2015. To date, 10 *mcr* variants, *mcr-1* to *mcr-10*, have been described. *mcr-8* was first identified in a carbapenem-resistant Klebsiella pneumoniae isolate (3). Unlike *mcr-1*, which is widely distributed in *Enterobacteriaceae*, *mcr-8* has only been detected in K. pneumoniae and two closely related species, Raoultella ornithinolytica and Klebsiella quasipneumoniae (4, 5). In recent years, due to the widespread dissemination of extended-spectrum β-lactamases and carbapenemases, Enterobacter cloacae has become the third most common *Enterobacteriaceae* leading to nosocomial infections (6). Currently, only *mcr-1*, *mcr-4*, *mcr-9*, and *mcr-10* have been detected in E. cloacae, mainly *mcr-9*. In this study, we report the emergence of *mcr-8* in E. cloacae and characterize the MDR E. cloacae clinical isolate phenotypically and genotypically.

E. cloacae clinical isolate SD21 was recovered from the sputum sample of a male patient suffering from chronic obstructive pulmonary disease at a tertiary hospital in Shandong, China. The isolate was initially identified using mass spectrometry and confirmed by next-generation sequencing. MICs of 15 antimicrobial agents were determined by the broth microdilution method. The MIC of tigecycline was interpreted according to the European Committee on Antimicrobial Susceptibility Testing (EUCAST) guidelines. And the interpretation of MICs of the remaining antimicrobials was based on the Clinical and Laboratory Standards Institute (CLSI) guidelines. This isolate exhibited a multidrug-resistant phenotype, with resistance to polymyxin B (>8 μg/mL), cefmetazole, ceftazidime, cefotaxime, cefepime, tigecycline, ciprofloxacin, amikacin, and aztreonam (Table S1). Conjugation assays were performed using the Escherichia coli J53 as the recipient strain (3), but no transconjugants were obtained after three assays. This result implied that the *mcr-8* may be on a nonconjugative genetic element, which is consistent with previous reports (7).

**FIG 1:**
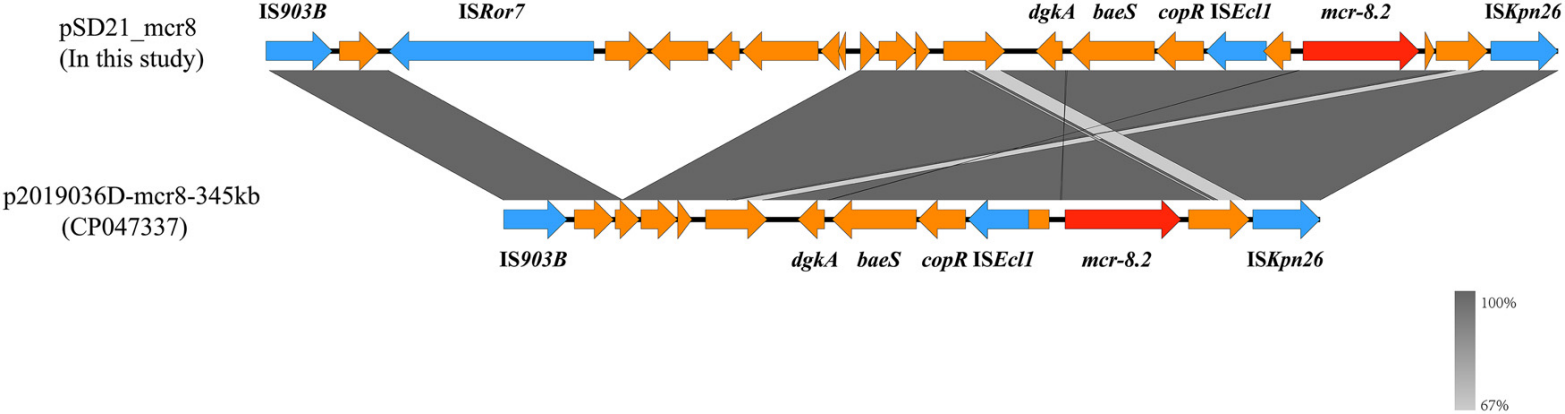
Comparison of the genetic environment of *mcr-8.2*. Linear alignment of genetic environments of pSD21_mcr8 and p2019036D-mcr8-345kb. Red arrows indicate *mcr-8.2*, blue arrows indicate mobile elements and yellow arrows indicate other proteins. Gray shading indicates homologous regions.

To investigate the genetic structure of *mcr-8*, the genome of SD21 was sequenced by short-read Illumina NovaSeq PE150 (Novogene, China) and long-read nanopore MinION (8). Whole-genome sequencing (WGS) results showed the presence of five circular DNA sequences, including one chromosome (CP093914) and four plasmids, namely pSD21_mcr8 (CP093916), pSD21_266kb (CP093915), pSD21_54kb (CP093917), and pSD21_4kb (CP093918). According to multilocus sequence typing, SD21 was assigned as ST1718. Notably, a *mcr-8* variant exhibiting 100% identity to *mcr-8.2* was identified in pSD21_mcr8 (100 852 bp). The plasmid pSD21_mcr8, belonging to IncFIA(HI1)/IncFII(K), harbored *mcr-8.2* within the genetic context IS*903B*-*orf*-IS*Ror7*-*orf*-*dgkA*-*baeS*-*copR*-IS*Ecl1*-*orf*-*mcr-8.2*-*orf*-IS*Kpn26* (Fig. 1). The *mcr-8.2* gene was flanked by insertion sequences IS*903B*, IS*Ror7*, and IS*Kpn26* which may play a role in its dissemination. The insertion of IS*Ecl1* between *copR* and *mcr-8.2* implied that IS*Ecl1* insertion was independent and unrelated to the mobilization of *mcr-8.2* (5). However, IS*Ecl1* may play a role in mobilization events in the future. The genetic structure around *mcr-8.2* in pSD21_mcr8 was highly consistent with that reported in K. pneumoniae, demonstrating that the *mcr-8.2* located region may have an ancestor from K. pneumoniae and disseminate among different pathogens. Online BLASTN comparison of pSD21_mcr8 showed that it had the highest similarity to p2019036D-mcr8-345kb (CP047337) from K. pneumoniae (Fig. S1), showing 99.91% identity and 100% coverage to p2019036D-mcr8-345kb. Besides, the lack of an intact *tra* region may be one of the aspects limiting the mobilization of pSD21_mcr8. However, the lack of *tra* operon did not prevent the mobilization of *mcr-8*-carrying plasmids between K. pneumoniae and E. cloacae. There could be other underlying mobilization mechanisms that mediate the dissemination of *mcr-8*-carrying plasmids in different species.

In addition to pSD21_mcr8, SD21 was found to harbor another resistance plasmid, designated pSD21_266kb. The pSD21_266kb with IncHI2/IncHI2A replicon type contained genes encoding resistance to β-lactams (*bla*_CTX-M-55_, *bla*_TEM-1B_), fosfomycin (*fosA3*), aminoglycosides (*aac(3)-IId*, *aph(3′)-Ia*, *aph*(6*)-Id*, *rmtB*, *aadA22*), macrolides (*mph*[A]), quinolones (*qnrS1*), sulfonamides (*sul3*), trimethoprim (*dfrA14*), tetracyclines (*tet*[A]), rifamycin (*ARR-2*), amphenicols (*floR*), and lincosamides (*lnu*[F]). Therefore, this plasmid enabled SD21 to exhibit resistance to multiple antimicrobial agents. The pSD21_266kb shared 99.99% identity with p16-6773.1 (CP039861.1) from Salmonella sp. (Fig. S2). The other two plasmids in SD21 did not harbor accessory resistance genes.

Given that no reports have described the existence of *mcr-8* in E. cloacae, identification of an E. cloacae isolate with *mcr-8.2* indicates the further dissemination of the *mcr-8* variant in different species. Furthermore, the *mcr-8.2*-bearing plasmid coexisted with a multidrug-resistant plasmid, which increased the difficulty of clinical treatment. In conclusion, we first reported an MDR E. cloacae strain with *mcr-8.2* isolated from a patient in China. We hypothesize that the *mcr-8.2*-located transferable genetic elements may be the genetic basis for the transmission of *mcr-8.2* among different species. Further research on epidemiology and transmission mechanism should be conducted to better understand the potential dissemination of *mcr-8*.

### Data availability.

The complete sequences of SD21 were deposited in the NCBI database with accession numbers CP093914, CP093915, CP093916, CP093917, and CP093918 under the BioProject PRJNA816615.
