# Supplementary material for: Emergence of Mobilized Colistin Resistance Gene mcr-8.2 in Multidrug-Resistant Enterobacter cloacae Isolated from a Patient in China
Source: Microbiol Spectr. 2022 Jun 21;10(4):e01217-22. doi: 10.1128/spectrum.01217-22 (PMC9430460; doi:10.1128/spectrum.01217-22)
Supplement: Supplemental file 1 — Supplemental material. Download spectrum.01217-22-s0001.pdf, PDF file, 0.4 MB [file spectrum.01217-22-s0001.pdf]

Supplemental Material

Table S1. Antimicrobial susceptibility profiles of strain SD21 (MICs µg/mL).

Strains	IPM	MEM	ETP	CMZ	CAZ	CTX	TZP	SCF	CAV	FEP	PB	TGC	CIP	AK	ATM
SD21	<1	<1	<2	>128	64	>128	<8/4	<8/4	<0.5/4	>64	>8	>4	>32	>128	64

Abbreviations: IMP, imipenem; MEM, meropenem; ETP, ertapenem; CMZ, cefmetazole; CAZ, ceftazidime; CTX, cefotaxime; TZP, piperacillin/tazobactam; SCF, cefoperazone/sulbactam; CAV, ceftazidime/avibactam; FEP, cefepime; PB, polymyxin B; TGC, tigecycline; CIP, ciprofloxacin; AK, amikacin; ATM, aztreonam.

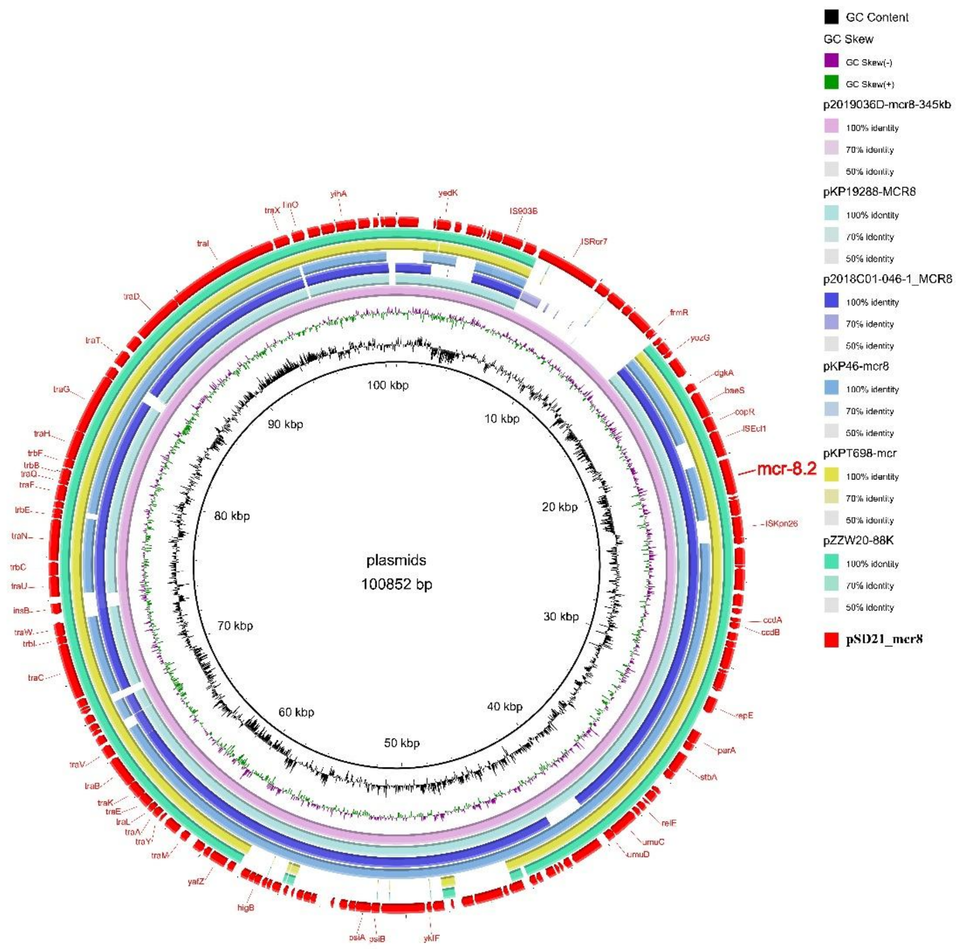

Figure S1. Comparative analysis of pSD21_mcr8 (this study) with p2019036D-mcr8-345kb (CP047337) and other similar plasmids. The selection criteria for similar plasmids were that the pSD21_mcr8 showed ≥ 99.00% identity and ≥ 80% coverage to the matched plasmids.

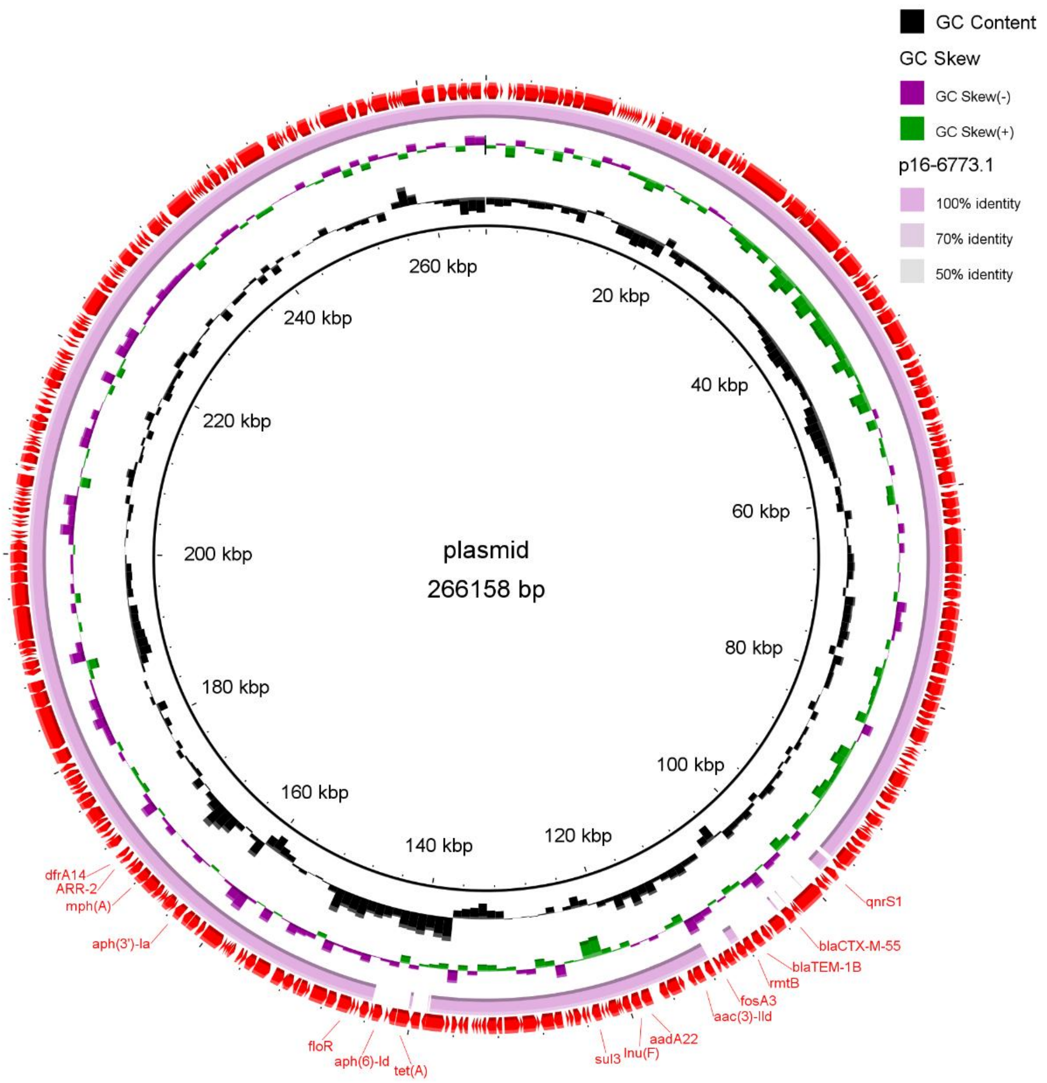

Figure S2. Comparative analysis of pSD21_266kb (this study) with p16-6773.1 (CP039861.1).
